# Global Prevalence of Severe Neonatal Jaundice among Hospital Admissions: A Systematic Review and Meta-Analysis

**DOI:** 10.3390/jcm12113738

**Published:** 2023-05-29

**Authors:** Udochukwu M. Diala, Fatima Usman, Duke Appiah, Laila Hassan, Tolulope Ogundele, Fatima Abdullahi, Katherine M. Satrom, Caitlin J. Bakker, Burton W. Lee, Tina M. Slusher

**Affiliations:** 1 Department of Paediatrics, Faculty of Clinical Sciences, College of Health Sciences, University of Jos, University of Jos Lamingo Campus, Jos 930232, Nigeria; 2 Department of Paediatrics, Faculty of Clinical Services, College of Health Sciences, Bayero University, Aminu Kano Teaching Hospital Campus, Kano 700006, Nigeria; 3 Department of Public Health, School of Population and Public Health, Faculty of Public Health, Texas Tech University Health Sciences Center, Lubbock, TX 79430, USA; 4 Department of Paediatrics, Faculty of Clinical Sciences, College of Medical Sciences, Ahmadu Bello University, Main Campus, Zaria 810211, Nigeria; 5 Department of Paediatrics, Obafemi Awolowo University Teaching Hospital, Ile-Ife 220005, Nigeria; 6 Department of Pediatrics, Faculty of Medical School, School of Medicine, University of Minnesota, University of Minnesota Twin Cities Campus, Minneapolis, MN 55455, USA; 7 Dr. John Archer Library and Archives, University of Regina, Regina, SK S4S 0A2, Canada; 8 National Institute of Health, Bethesda, MD 20892, USA; 9 Hennepin Healthcare, Minneapolis, MN 55415, USA

**Keywords:** neonatal, jaundice, hyperbilirubinemia, global prevalence

## Abstract

Evidence regarding the adverse burden of severe neonatal jaundice (SNJ) in hospitalized neonates in resource-constrained settings is sparse. We attempted to determine the prevalence of SNJ, described using clinical outcome markers, in all World Health Organization (WHO) regions in the world. Data were sourced from Ovid Medline, Ovid Embase, Cochrane Library, African Journals Online, and Global Index Medicus. Hospital-based studies, including the total number of neonatal admissions with at least one clinical outcome marker of SNJ, defined as acute bilirubin encephalopathy (ABE), exchange blood transfusions (EBT), jaundice-related death, or abnormal brainstem audio-evoked response (aBAER), were independently reviewed for inclusion in this meta-analysis. Of 84 articles, 64 (76.19%) were from low- and lower-middle-income countries (LMICs), and 14.26% of the represented neonates with jaundice in these studies had SNJ. The prevelance of SNJ among all admitted neonates varied across WHO regions, ranging from 0.73 to 3.34%. Among all neonatal admissions, SNJ clinical outcome markers for EBT ranged from 0.74 to 3.81%, with the highest percentage observed in the African and South-East Asian regions; ABE ranged from 0.16 to 2.75%, with the highest percentages observed in the African and Eastern Mediterranean regions; and jaundice-related deaths ranged from 0 to 1.49%, with the highest percentage observed in the African and Eastern Mediterranean regions. Among the cohort of neonates with jaundice, the prevalence of SNJ ranged from 8.31 to 31.49%, with the highest percentage observed in the African region; EBT ranged from 9.76 to 28.97%, with the highest percentages reported for the African region; ABE was highest in the Eastern Mediterranean (22.73%) and African regions (14.51%). Jaundice-related deaths were 13.02%, 7.52%, 2.01% and 0.07%, respectively, in the Eastern Mediterranean, African, South-East Asian and European regions, with none reported in the Americas. aBAER numbers were too small, and the Western Pacific region was represented by only one study, limiting the ability to make regional comparisons. The global burden of SNJ in hospitalized neonates remains high, causing substantial, preventable morbidity and mortality especially in LMICs.

## 1. Introduction

Severe neonatal jaundice (SNJ) in a neonate may manifest as acute bilirubin encephalopathy (ABE) [1] with a range of symptoms including difficulty feeding, tone abnormalities, abnormal cry and the kernicteric facies [2] scored using the bilirubin-induced neurological dysfunction (BIND) score or modified BIND [3,4]. Persistent abnormalities which are now known as the Kernicterus Spectrum Disorder (KSD) [1], occur in 70% of survivors beyond the neonatal period including choreo-athetoid cerebral palsy, deafness, speech and language processing disorders, enamel dysplasia, and learning difficulties [5,6,7].

The Global Burden of Disease study ranks SNJ among the top 5–10 causes of neonatal deaths in countries with the highest number of neonatal deaths [8]. Previous attempts at providing global and regional estimates of SNJ burden have been challenged by limited data. Bhutani et al. estimated 481,000 global cases of SNJ among term/near-term neonates with 114,000 deaths and 75,000 of survivors developing kernicterus [9]. These figures derived using mathematical models with limited data have limitations inherent in such estimates. A previous population-based systematic review and meta-analysis including some authors in our current team (TS, DA, BL) reported a pooled incidence of SNJ at 244 per 100,000 live births [10]. A major drawback of this review was the disproportionate representation of high-income countries with lesser burden of disease. Several studies have suggested that the African and Asian regions have the highest burden of disease [9,10,11]. Factors responsible for these regional burdens include the high prevalence of glucose-6-phosphate deficiency (G6PD) deficiency, late presentation due to the high incidence of out-of-hospital births, inability of caregivers to promptly identify jaundice, caregivers’ decision to seek alternative treatments; lack of or ineffective phototherapy and unavailable or unreliable access to bilirubin estimations [11]. Unfortunately, most data on SNJ in low-resource countries is hospital-based without true population-based data making the actual burden of SNJ unknown. However, this review of hospital-based data covers a wider representation of literature from diverse countries to ascertain, though still imperfect, the burden in low and lower-middle-income countries (LMICs). Our intent is to be the first comprehensive, current systematic review and meta-analysis that provides rigorous, worldwide appraisal of SNJ for all neonatal hospital admissions which included adverse clinical outcomes seen in SNJ, to compare regional geographic differences, and to provide representation from low/lower-resource areas. These data are critical not only to meet the global Sustainable Development Goals (SDGs) but also to assist in identifying region-specific strategies to decrease disability-adjusted life years (DALYs) from KSD morbidities [12].

## 2. Materials and Methods

### 2.1. Criteria for Article Inclusion

We included hospital-based studies that had neonatal hospital admissions for any cause and provided information about at least one clinical marker of SNJ, including number of exchange blood transfusions (EBTs); ABE; abnormal brainstem audio-evoked response (aBAER); or jaundice-related death. No patients or members of the public were involved in any way, and data were from published sources; therefore, investigational review board or ethics committee approval was not needed.

### 2.2. Criteria for Article Exclusion

Articles were excluded if (1) the entire data collection period was prior to 1997 or later than 2020, (2) sample size was <10, (3) period of data collection was not defined, (4) jaundice was conjugated, from metabolic or neonatal liver disease, (5) EBT was done for conditions unrelated to SNJ, (6) non-English, (7) non-neonatal, (8) publication type was a review article, questionnaire or survey or study design was case-control or experimental study on a subset of neonates with jaundice, and (9) missing critical data (total number of neonatal admissions). In the case of missing data, we (FA, TS) contacted the authors for further information and excluded the article if the requested information was not supplied. Magnetic resonance imaging (MRI) was excluded due to no returned results.

### 2.3. Outcome Definition

Our primary outcome was the prevalence of SNJ in hospitalized neonates (both inborn and outborn) clinically defined as having at least one clinical indicator of SNJ noted above. We also looked at the prevalence of SNJ in hospitalized jaundiced neonates again using clinical markers noted above. LMIC status was defined using the 2020 World Bank Criteria [13].

### 2.4. Search Criteria

**Figure 1:**
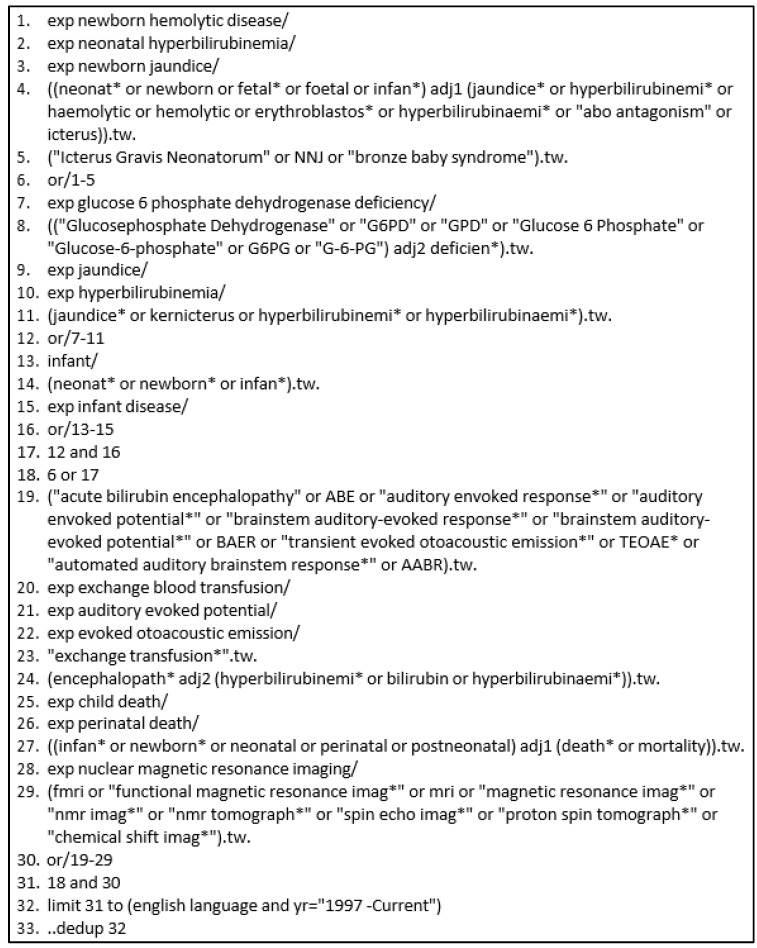
Search Protocol: Embase Classic + Embase via Ovid. * is a truncation symbol.

We conducted a comprehensive search including both natural language and controlled vocabulary terms to reflect concepts of a neonatal population and jaundice, including both serum bilirubin and clinical indicators. The search was conducted across five databases: Ovid Medline, Ovid Embase, Cochrane Library via Wiley, African Journals Online, and Global Index Medicus (Figure 1). This search strategy was translated across the different databases to ensure appropriate use of the available controlled vocabulary and unique search functionality. The search was conducted in June 2018 and updated in September 2020. The protocol was registered in Prospero (CRD42018100214). A PRISMA checklist was completed (Table S1).

### 2.5. Data Extraction

Articles were screened using the Rayyan software for systematic reviews [14]. One author (CB) conducted the literature search and uploaded all eligible abstracts onto the software. Three groups of reviewers comprising of two authors per group [group A (UD and TO), group B (FU and LH) and group C (KS and FA)] were involved in the literature review.

**Figure 2:**
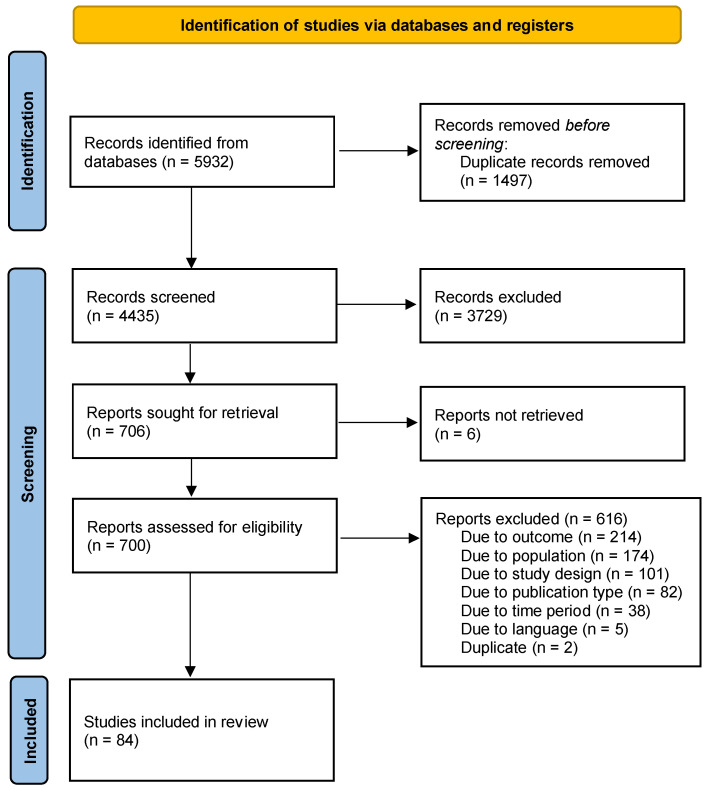
PRISMA flow diagram showing the outcome of database searches and the process of selection of included studies.

During screening, each group member independently assessed the allocated articles’ titles and abstracts for eligibility. Discrepancies were resolved via group dialog and, when necessary, a third author (TS) acted as an arbitrator. This was followed by full-text screening with reasons for exclusion recorded (Figure 2).

Data extraction forms were developed, piloted, and refined. Data were extracted using Qualtrics^®^, including citation information, country and World Health Organization (WHO) region, study duration, total number of neonatal admissions and neonatal jaundice (NNJ) admissions, gestational age (all, only term, only near-term, term and near-term combined, only preterm or unspecified), how jaundice was determined (clinical definition or using bilirubin assay), markers of SNJ: number of EBTs, aBAER, and reported jaundice-related deaths. Microsoft Excel spreadsheet was used to collect data for analysis. Table 1 shows the profile of articles selected for the meta-analysis.

### 2.6. Risk of Bias (Quality) Assessment

Each article was scored based on five parameters that were modified from those used in a prior population-based study also using clinical parameters to assess the burden of disease from SNJ [11]. Scoring was in line with recommendations by the modified quality assessment tool for systematic reviews of observational studies (QATSO) scoring system [98]. These included: (1) if the sampling method was representative of the target population i.e., covered the whole nursery population (scored 3), term and near-term only (scored 2), premature or term only (scored 1); (2) the method used to define jaundice, categorized based on bilirubin assay (scored 2), visual clinical assessment (scored 1) or not stated (scored 0); (3) whether the study excluded any of the following conditions: Glucose -6 Phosphate Dehydrogenase deficiency, ABO incompatibility, Rhesus incompatibility or sepsis and was grouped as yes (scored 0) or no (scored 1); (4) if total number of SNJ cases was reported and classed as yes (scored 1) or no (scored 0); and (5) whether clinically significant jaundice was clearly defined in the Methods section (including use of AAP/NICE guidelines) and classified as yes (scored 1) or no (scored 0). Each study’s quality was judged based on aggregate points, with a maximum obtainable score of 10, and classified as “good quality” (7–10 points), “fair quality” (4–6 points) and “poor quality” (0–3 points).

### 2.7. Statistical Analysis

The summary estimate for the meta-analysis was prevalence/proportion, which was transformed using Freeman–Tukey double arcsine transformation to enable them to correspond to probabilities under the standard normal distribution and enhance significance testing [99]. The double transformations adequately addressed issues of variance instability as well as confidence intervals (CIs) of proportions falling outside the possible range of 0 to 1 for binomial data [100]. Pooled estimates were calculated using DerSimonian and Laird’s random-effects method, weighting individual study estimates using the inverse of the variance of their transformed proportion as study weight, with their 95% CI determined using the Clopper–Pearson exact binomial method [101]. Statistical heterogeneity among studies was assessed using Cochran’s Q test and I^2^ with a *p*-value of <0.10. The I^2^ quantifies the proportion of the dispersion that is real and not spurious [102]. Possible sources of heterogeneity were also explored via subgroup analysis.

Additionally, mixed-effects meta-regression analysis was used to determine whether study-level covariates, such as publication year, country-level income and methodological domains for assessing study quality, explained some of the observed between-study heterogeneity. A formal test of publication bias was assessed using Begg’s adjusted rank correlation [102] and Egger’ regression asymmetry tests [103], as well as through visual interpretation of funnel plots. Analyses were conducted using R software (version 4.3.0; R Foundation for Statistical Computing, Vienna, Austria). 

## 3. Results

The electronic databases search identified 4436 distinct articles (after removing 1497, duplicates) (Figure 2). An additional 3729 articles were excluded after reviewing titles and abstracts. Seven hundred and six (706) articles were selected for full article review and 700 (99%) were retrieved and reviewed. The remaining six articles were unavailable from any source that we could access. Eighty-four hospital-based studies involving a total of 2,210,043 neonatal admissions and 5986 neonates with at least one marker of SNJ were included in the meta-analysis (Table 1) [6,15,16,17,18,19,20,21,22,23,24,25,26,27,28,29,30,31,32,33,34,35,36,37,38,39,40,41,42,43,44,45,46,47,48,49,50,51,52,53,54,55,56,57,58,59,60,61,62,63,64,65,66,67,68,69,70,71,72,73,74,75,76,77,78,79,80,81,82,83,84,85,86,87,88,89,90,91,92,93,94,95,96,97].

Sixty-four (76.19%) of the studies were conducted in LMICs (low (5) and lower-middle (59)), including 43 (51.19%) from the African region and 1 (1.19%) from the Eastern Mediterranean region (Table 1). Fourteen (16.67) were from upper-middle income countries. Six (7.1%) of the articles were from high-income countries. Both preterm and term neonates were included in 54 (64.2%) studies. Half (43/84) of the studies were adjudged to be of high quality (Table 2).

NNJ was included in the diagnoses for 21.99% (95% CI: 18.42–25.78%) of all neonatal admissions in articles included in our review with a significant difference (*p* < 0.001) between WHO regions ranging from 30.61% (95% CI 22.19–39.74%) in South-East Asia, 20.39% (95% CI 11.73–30.70%) in Europe, 20.10% (95% CI 16.06–24.47%) in Africa, 16.66% (95% CI 4.90–33.54%) in the Eastern Mediterranean, 13.61% (95% CI 2.83–29.34%) in the Americas to 2.55% (95% CI 2.37–2.75%) in the Western Pacific region represented by only one article (Table 3).

**Figure 3:**
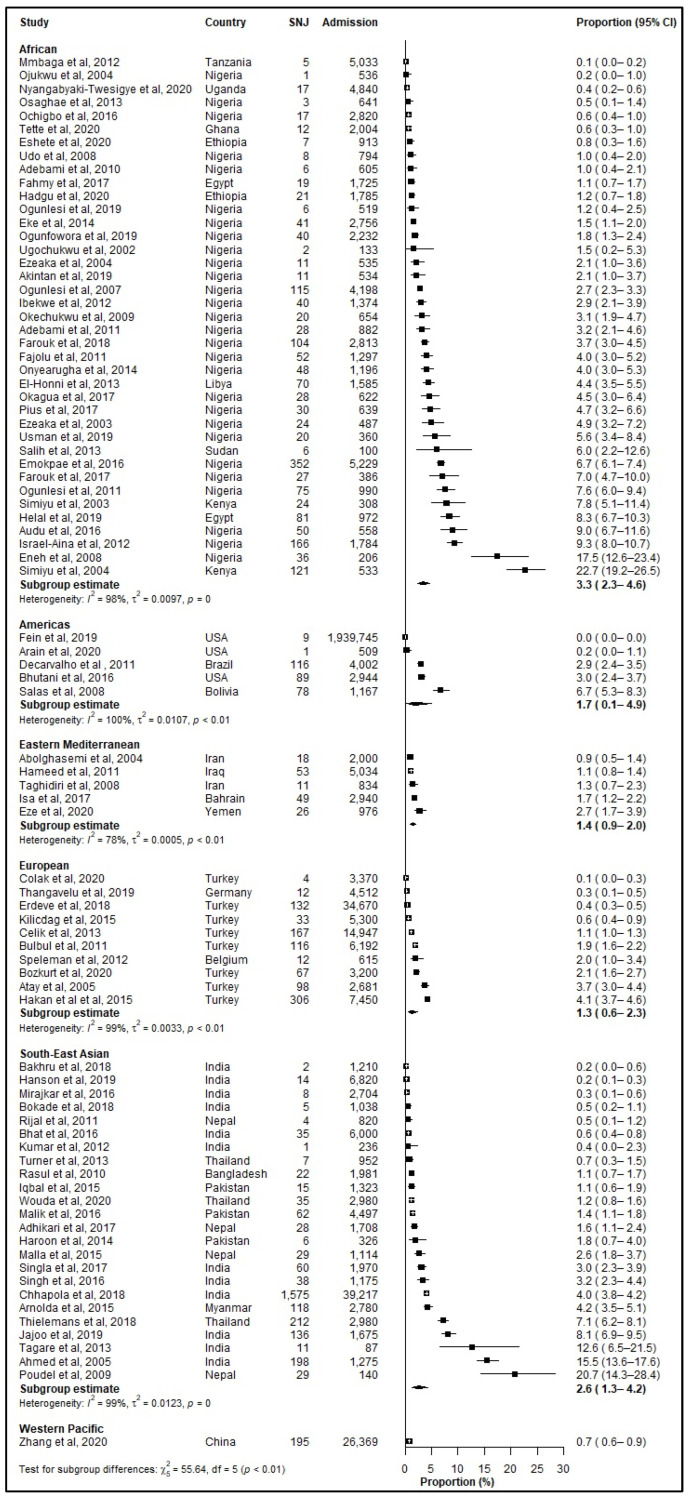
Prevalence (%) of severe neonatal jaundice (SNJ) among hospitalized neonates according to World Health Organization (WHO) regions. CI: Confidence interval; SNJ: Severe Neonatal Jaundice; References: [6,15,16,17,18,19,20,21,22,23,24,25,26,27,28,29,30,31,32,33,34,35,36,37,38,39,40,41,42,43,44,45,46,47,48,49,50,51,52,53,54,55,56,57,58,59,60,61,62,63,64,65,66,67,68,69,70,71,72,73,74,75,76,77,78,79,80,81,82,83,84,85,86,87,88,89,90,91,92,93,94,95,96,97].

The prevalence of SNJ amongst all neonatal admissions (Figure 3) varied significantly across WHO regions (*p* < 0.001) with the African region reporting highest prevalence (3.34%, 95% CI: 2.28–4.57%), followed by the South-East Asian region (2.58%, 95% CI: 1.33–4.22) and the Americas (1.73%, 95% CI: 0.14–4.92) [Table 4].

**Figure 4:**
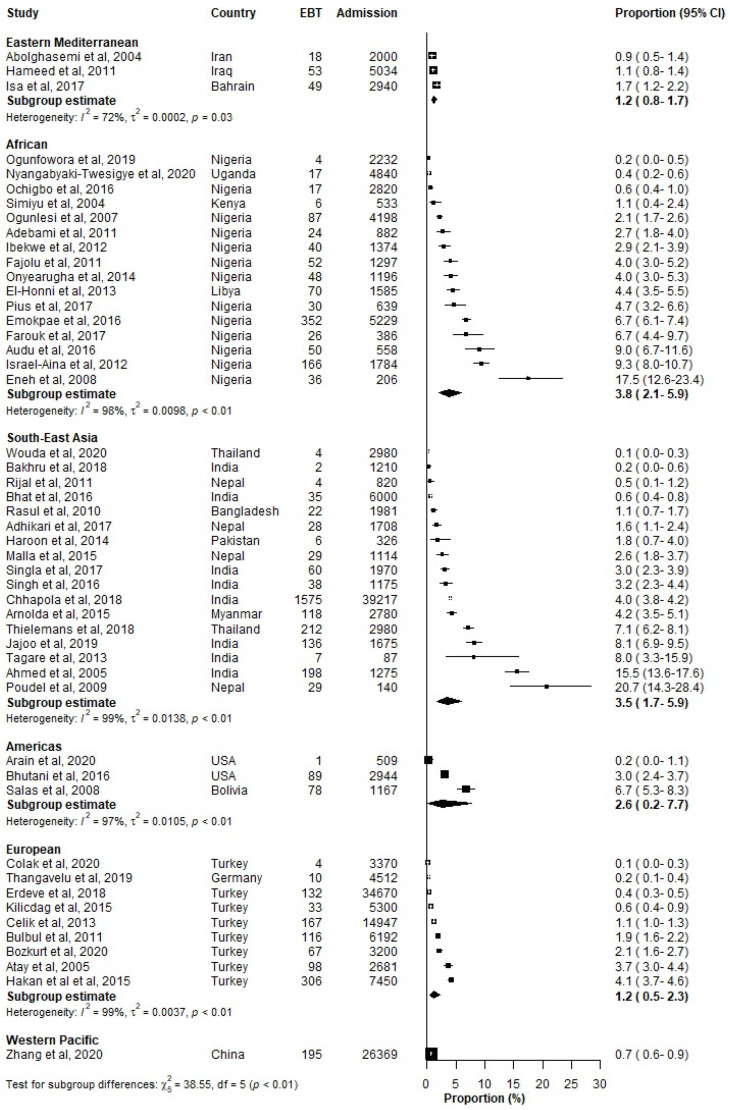
Prevalence (%) of severe neonatal jaundice (SNJ) with exchange transfusions (EBT) among hospitalized neonates according to WHO regions. CI: Confidence interval; EBT: Exchange Blood Transfusion; References: [15,17,18,19,21,22,23,24,25,26,27,29,30,31,32,33,36,37,38,39,46,47,49,51,53,55,57,58,59,60,62,66,67,68,69,75,77,78,79,80,82,83,84,85,86,88,91,92,96,97].

The prevalence of EBT among all neonates was the highest in the African region (3.81%, 95% CI: 2.14–5.92%), followed by the South-East Asian region (3.50%, 95% CI: 1.69–5.90%) (Table 4, Figure 4). Among jaundiced neonates, significant regional differences also existed (*p* < 0.001), with the African region reporting the highest prevalence of EBT at 21.42% (95% CI: 11.03–34.07) (Table 5).

**Figure 5:**
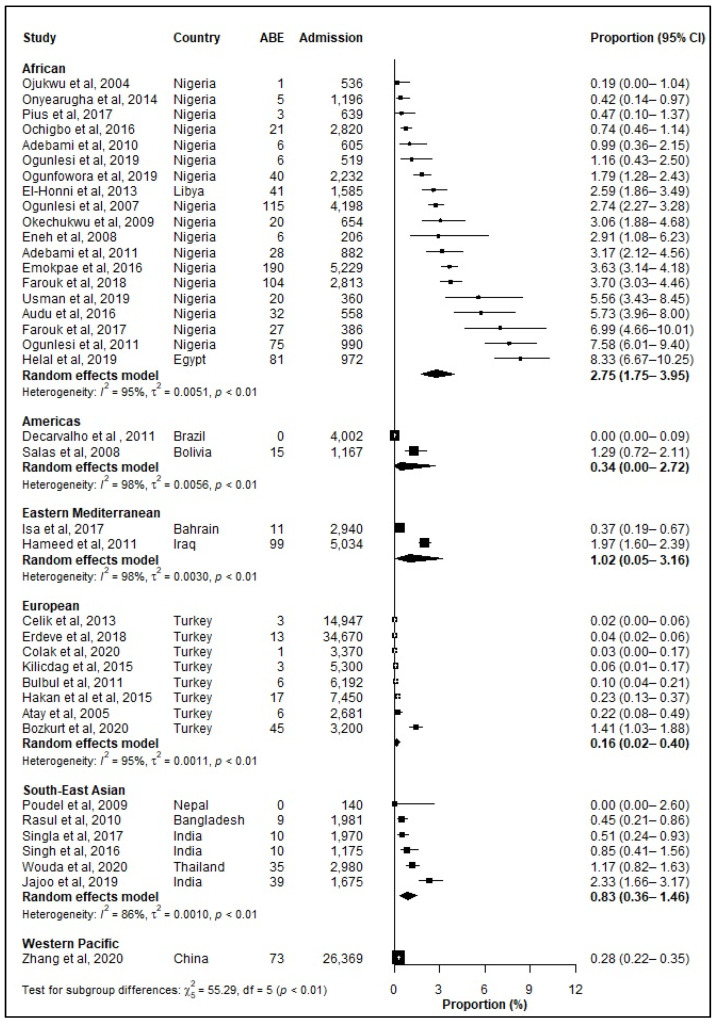
Prevalence (%) of severe neonatal jaundice (SNJ) with acute bilirubin encephalopa-thy/kernicterus (ABE) among hospitalized neonates according to WHO regions. ABE: Acute Bilirubin Encephalopathy; CI: Confidence interval. References: [6,16,17,23,24,30,31,33,36,37,38,39,47,48,50,51,57,60,67,69,70,72,75,77,78,79,82,85,86,87,91].

The prevalence of ABE among hospitalized neonates varied by WHO regions with the highest prevalence reported for the African region 2.75% (95% CI: 1.75–3.95%) (Figure 5, Table 4). ABE in jaundiced neonates was highest in the Eastern Mediterranean (22.73%) reporting the highest prevalence of ABE followed by (Table 5).

The highest proportion of jaundice-related deaths among all neonates was 1.49% (95% CI: 0.85–2.28%) in the African region (Table 4). This increased to 7.52% (95% CI: 4.95–10.56%) in neonates with jaundice (Table 5). The Eastern Mediterranean region was next with 1.24% (95% CI: 0.00–4.48%) in all neonates and increased to 13.02% (95% CI: 9.64–16.81%) in neonates with jaundice (Table 4 and Table 5).

**Figure 6:**
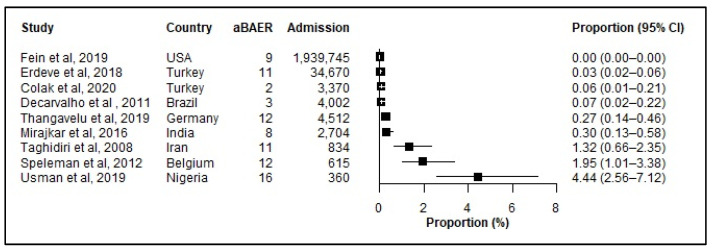
Prevalence (%) of abnormal Brainstem Auditory Evoked Response (aBAER) among hospitalized neonates. aBAER: abnormal Brainstem Auditory Evoked Response (aBAER); CI: Confidence interval; References: [6,33,34,39,45,64,87,89,91].

**Figure 7:**
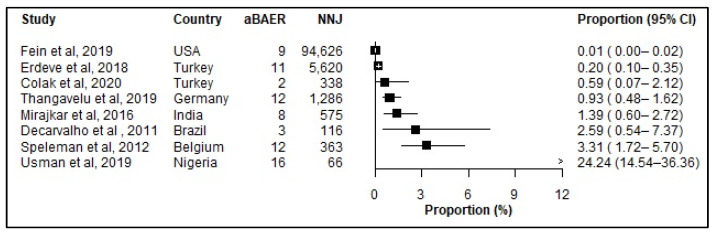
Prevalence (%) of abnormal Brainstem Auditory Evoked Response (aBAER) among neonates admitted with jaundice. aBAER: abnormal Brainstem Auditory Evoked Response (aBAER); CI: Confidence interval; References: [6,33,34,39,45,64,87,89,91].

Only nine studies reported aBAERs, making the reported results likely a gross underestimate (Figure 6). For comparison, eight studies reported aBAERS among neonates admitted with jaundice (Figure 7).

**Figure 8:**
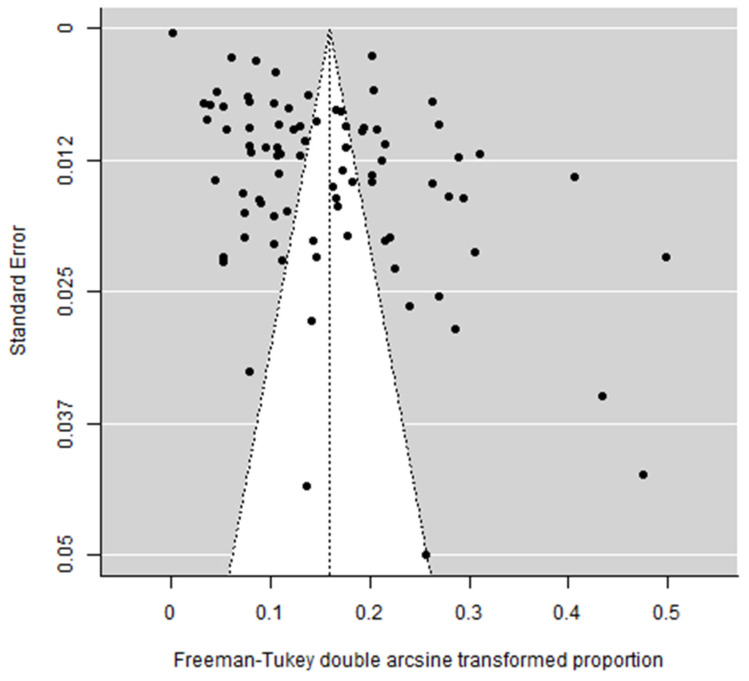
Funnel plot of studies included in the meta-analysis. The unshaded triangle represents the region within which 95% of studies would be expected to lie to lie if the studies are all estimating the same underlying effect. References: [6,15,16,17,18,19,20,21,22,23,24,25,26,27,28,29,30,31,32,33,34,35,36,37,38,39,40,41,42,43,44,45,46,47,48,49,50,51,52,53,54,55,56,57,58,59,60,61,62,63,64,65,66,67,68,69,70,71,72,73,74,75,76,77,78,79,80,81,82,83,84,85,86,87,88,89,90,91,92,93,94,95,96,97].

There was evidence of potential publication bias influencing the reporting of prevalence of SNJ. Studies that report a lower proportion of neonates with SNJ were less likely to be published. The funnel plot appeared largely asymmetrical (Figure 8) with empirical evidence supporting this observation (Begg test *p* < 0.001, Egger’s bias = 13.0, *p* < 0.001).

Of the six methodological domains included (5 domains used for assessing study quality and type of study facility), only facility type showed significant differences in estimates of the prevalence of SNJ in subgroup analysis (Table 6). In meta-regression analysis, publication year (<0.001), country income level (*p* = 0.009), representativeness of the sample to the target population (*p* = 0.04), and type of healthcare facility (*p* = 0.001) significantly explained 17.00% of the between-study heterogeneity in the observed prevalence of SNJ (Table 7). 

A one-year increase in publication year was found to predict a decrease in prevalence of SNJ by 0.6% (coefficient: −0.006 [95% CI: −0.010, −0.002]), indicating more recent studies tended to publish lower prevalence for SNJ compared to earlier published studies. Upper-middle-, lower-middle-, and low-income countries all had a prevalence of SNJ that was 4.10%, 8.70% and 3.60% higher than the prevalence of SNJ reported among high-income countries. Studies conducted on all neonates or term and near-term neonates had 6.20% and 7.50% lower prevalence than studies that only included term only or preterm only respectively. The prevalence of SNJ in tertiary/referral hospitals was 7.0% higher (coefficient: 0.070 [95% CI: 0.031, 0.109]) than studies that did not report type of healthcare facility. In multivariable meta regression analysis, year of publication, income level of country, sample representative of target population (whether it included term, preterm or whole neonate population), method used to define jaundice, study having reported total number of NNJ cases, whether clinically significant jaundice was clearly defined or not together explained 58% of the variation in SNJ prevalence across countries.

## 4. Discussion

Our data demonstrate that adverse clinical outcomes of SNJ remains a significant public health concern in LMICs. It continues to be a leading cause of neonatal admissions and death. SNJ contributes substantially to neonatal mortality worldwide, with the highest burden in the African (1.49%) and South-East Asian (0.82%) WHO regions. Our study highlights the global prevalence of SNJ with ranges varying from 3.34% in the African and 2.58% in the South-East Asian regions to 1.73%, 1.42%, 1.31% and 0.74% in the Americas, Eastern Mediterranean, European and Western Pacific regions. SNJ is associated with a substantial risk of long-term disability [2,8,9]. Of note, the prevalence declined slowly over time by 0.6% per year.

Focusing only on those with NNJ, our data show a prevalence of SNJ among this cohort ranging between 8.3% and 31.4%, with the highest burden of disease in the Western Pacific and African regions. However, the Western Pacific region was only represented by one report from China (upper middle-income) and the America’s had only five studies [USA-high-income (*n* = 3), Brazil-upper middle-income (*n* = 1), Bolivia-lower middle-income (*n* = 1)]. Despite efforts to find worldwide data there is still selection bias due to underreporting with only 27 of the 195 official countries providing any data at all.

Although still unevenly distributed with many countries without data, our review more accurately represents the global burden of SNJ than previous studies/reviews have done with 64 (76.19%) of the articles from LMICs and an additional 14 articles (16.67%) from upper-middle-income countries. This is a stark contrast to the previous population-based systematic review and meta-analysis, where 76% of the included studies were from high-income countries and, thus, much less representative of the actual income distribution globally than this present study [10]. Our current work included 84 studies representative of all WHO regions, including more country diversity and income levels within most regions. 

Additionally, most articles included in our review studied both term and preterm populations. Important because preterm infants have a higher prevalence of NNJ and a higher risk of neurological damage at lower bilirubin levels [27,104]. Of note, studies that included only preterm neonates reported a higher prevalence of SNJ than other studies, however, this difference did not attain statistical significance; an observation differing from other reports [27,105]. 

This review also reported a higher prevalence of SNJ in higher-tier health facilities when compared to primary and secondary health facilities, possibly because SNJ is usually managed in higher-tier facilities due to these facilities having more phototherapy devices and manpower [105]. The actual burden is likely underreported as many neonates do not reach tertiary centers in LMICs.

The current review highlights that NNJ is noted in 21.99% of all neonatal admissions across WHO regions in the studies included in our review, consistent with prior studies [23,43,82]. Of all neonatal admissions with jaundice, those that had clinical evidence of severe disease ranged from 8.31–31.49% with variability across regions with areas with higher prevalence in regions where neonates often present late to the hospital, likely attributable to previously identified factors [8,106,107]. 

Striking differences persist between WHO regions for individual SNJ markers again with wide ranges for both ABE and percentages of neonates requiring EBTs. Many complications are likely underreported due to the lack of follow-up and/or the ability to perform specialized testing including BAER or MRI. For these reasons, along with the lack of representation of many countries, we expect that these data significantly underestimate the true burden of severe disease. With known effective treatment strategies, including intensive phototherapy and EBT, likely coupled with maternal education, early timely diagnosis, and treatment [106,108,109], these complications are preventable in almost all neonates. 

Our study also looked at other factors potentially associated with SNJ. With the meta-regression, three factors—publication year, type of study facility and country income level accounted for 58% of the heterogeneity. More recent studies tended to report a lower prevalence of SNJ which may reflect modest gains in recently introduced national programs such as the “Every Newborn Action Plan (ENAP)” focusing on newborn risk assessment, identification of cases with prompt referrals, maternal education and postnatal visits and having the potential of reducing behavioral factors that contribute to SNJ [110]. 

Study limitations include the continued underrepresentation of several regions/countries in this data set and the decision to limit the search to English only based on the lack of any population-based data in other languages in the previous population-based review. Inability to accurately ascertain place of birth and uniformly determine how many neonates were readmissions versus admissions from outside of healthcare facilities. Bilirubin levels were not required because bilirubin levels are not uniformly available in all hospitals and definitions for severe hyperbilirubinemia vary widely. Another limitation of this review is in the observed high degree of heterogeneity of pooled prevalence which is not unexpected for prevalence studies with marked methodological differences. Though we tried to deal with the high heterogeneity by looking at the effect of design, year and population characteristics in the meta-regression, we still found significant heterogeneity. Despite the high heterogeneity, it is clear that the burden of disease remains high, with a much higher proportion of the disease in LMICs. This does not alter the significance of our study as it is a representation of available research done thus far in hospitalized neonates. We also failed to link prevalence of SNJ in this review to the predicted prevalence of long-term sequelae. A disappointing limitation is the small number of studies from three of WHO regions (Americas, Eastern Mediterranean, and Western Pacific), decreasing generalizability of the findings in these regions. Of note, the Americas did have stronger representation in the previous population-based review and did not have a high prevalence of SNJ [10]. This study could have been potentially strengthened by adding additional weighting based on the prevalence of known factors, such as G6PD deficiency, Rhesus disease and neonatal sepsis, which vary among different WHO regions in neonates with SNJ. This additional weighting should be included in future systematic reviews and meta-analyses to help determine the global burden of SNJ. A strength of this study is that it included global data, and most of articles analyzed were adjudged to be of high quality which strengthens the validity of our findings. The relatively high representation from the African, European, and South-East Asian regions and middle-income countries enhances generalizability of our findings to these regions. Overall, this review had better representation than our previous population-based study although attempts to get population-based data should continue.

As we highlighted in the limitations section above, our data demonstrated high heterogeneity but, despite that limitation, provides the best representation of the burden of disease, especially in LMICs/LICs available at this time. Country and regional registries and population data are urgently needed but only largely available in a few high-income countries globally [10]. Should true population-based data become widely available, they will provide more robust and generalizable data. However, if we wait for that population data to come, it will likely be years if not decades before important stakeholders, such as the WHO and United nations Children’s Fund (UNICEF), move SNJ to the top of their list of global neonatal priorities. Using mathematical modeling, Bhutani et al. [9] predicted that in 2010, there were 240 million infants at risk for neonatal hyperbilirubinemia-related adverse outcomes, and 750,000 with KSD. With increasing populations in Africa and other LMICs where the burden of SNJ is highest, these estimates will increase if mitigation factors are not implemented. More studies are also needed that factor in the medical standards and risks for developing jaundice in each country along country and even within-country regional-specific guidelines based on the risks and treatment available within a given country or region. As highlighted in American Academy of Pediatrics (AAP) 2022 guidelines, and also highlighted in a recent perspective piece, LMICs need to base treatment on their own risks and resources [111,112]. Using AAP guidelines would potentially lead to a substantially higher burden of both ABE and KSD than we currently see in LMICs.

## 5. Conclusions

SNJ remains an important contributor to neonatal morbidity and mortality, especially in the African and South-East Asian regions. As we work towards the SDGs of improving neonatal mortality and the goal of decreasing morbidity, SNJ needs to be addressed as a preventable cause of both-most effectively addressed with a package approach which includes maternal, community and healthcare provider education; country specific guidelines based on risk and resources; accurate reliable low-cost methods of screening and diagnosis including not only bilirubin levels but also blood grouping and Rhesus as well as G6PD screening, effective phototherapy, capabilities to do safe EBT’s when indicated and comprehensive follow-up and treatment for all children with KSD.

## Figures and Tables

**Table 1:** Characteristics of studies included in Meta-analysis [6,15,16,17,18,19,20,21,22,23,24,25,26,27,28,29,30,31,32,33,34,35,36,37,38,39,40,41,42,43,44,45,46,47,48,49,50,51,52,53,54,55,56,57,58,59,60,61,62,63,64,65,66,67,68,69,70,71,72,73,74,75,76,77,78,79,80,81,82,83,84,85,86,87,88,89,90,91,92,93,94,95,96,97].

	WHO Region	Country	Admission	NNJ	Gestation	EBT	aBAER	ABE	Deaths	SNJ	Risk of Bias	Ref #
Abolghasemi H et al., 2004	Eastern Med	Iran **	2000	283	All	18				18	7	[15]
Adebami OJ et al., 2010	African	Nigeria **	605	89	All			6		6	5	[16]
Adebami OJ et al., 2011	African	Nigeria **	882		All	24		28	9	28	6	[17]
Adhikari S et al., 2017	SE Asian	Nepal **	1708	662	All	28				28	5	[18]
Ahmed et al., 2005	SE Asian	India **	1275	305	All	198				198	7	[19]
Akintan PE et al., 2019	African	Nigeria **	534	158	Only term				11	11	5	[20]
Arain Y et al., 2020	Americas	USA ^++^	509		Preterms only	1				1	6	[21]
Arnolda G et al., 2015	SE Asian	Myanmar **	2780	989	All	118				118	8	[22]
Atay E et al., 2005	European	Turkey ^+^	2681	624	Term	98		6		98	5	[23]
Audu LI et al., 2016	African	Nigeria **	558	123	Term-near term	50		32	16	50	7	[24]
Bakhru V DR et al., 2018	SE Asian	India **	1210	121	Term and near-term	2				2	7	[25]
Bhat P et al., 2016	SE Asian	India **	6000	406	Term-near term	35				35	7	[26]
Bhutani V et al., 2016	Americas	USA ^++^	2944	677	Term-near term	89				89	7	[27]
Bokade C et al., 2018	SE Asian	India **	1038	101	All				5	5	5	[28]
Bozkurt O et al., 2020	European	Turkey ^+^	3200	115	Term and near-term	67	45			67	7	[29]
Bulbul A et al., 2011	European	Turkey ^+^	6192	782	Term-near term	116		6	1	116	7	[30]
Celik HT et al., 2013	European	Turkey ^+^	14,947	4906	All	167		3		167	8	[31]
Chhapola V et al., 2018	SE Asian	India **	39,217		Not specified	1575				1575	4	[32]
Colak R CS et al., 2020	European	Turkey ^+^	3370	338	Not specified	4	1	2	12	4	6	[33]
de Ccarvalho et al., 2011	Americas	Brazil ^+^	4002	116	Term-near term		3			116	8	[34]
Eke CV et al., 2013	African	Nigeria **	2756		All				41	41	7	[35]
El-Honni MS et al., 2013	Eastern Med	Libya ^+^	1585	400	Term	70		41	2	70	6	[36]
Emokpae AA et al., 2016	African	Nigeria **	5229	1118	All	352		190	61	352	8	[37]
Eneh AU et al., 2008	African	Nigeria **	206	44	All	36		6	2	36	8	[38]
Erdeve O et al., 2018	European	Turkey ^+^	34,670	5620	Term and near-term	132	13	11	2	132	7	[39]
Eshete A et al., 2020	African	Ethiopia *	913	52	All				7	7	5	[40]
Eze P et al., 2020	Eastern Med	Yemen *	976	183	All				5	5	5	[41]
Ezeaka C et al., 2004	African	Nigeria **	487	141	All				24	24	7	[42]
Ezeaka C et al., 2005	African	Nigeria **	535	104	Preterms only				11	11	5	[43]
Fahmy N et al., 2017	Eastern Med	Egypt **	1725	647	All				19	19	6	[44]
Fein EH et al., 2019	Americas	USA ^++^	1,939,745	94,626	Only term		9		9	9	6	[45]
Fajolu IB et al., 2011	African	Nigeria **	1297		All	52				52	4	[46]
Farouk Z et al., 2017	African	Nigeria **	386	100	All	26		27	11	27	8	[47]
Farouk Z et al., 2018	African	Nigeria **	2813	551	All			104	33	104	8	[48]
Hadgu FB et al., 2020	African	Ethiopia *	1785	247	All				21	21	5	[49]
Hakan N et al. et al., 2015	European	Turkey ^+^	7450	1862	All	306		17	3	306	7	[50]
Hameed NN et al., 2014	Eastern Med	Iraq ^+^	5034	162	Term-near term	53		99	19	53	7	[51]
Hanson C et al., 2019	SE Asian	India **	6820	1513	All				14	14	5	[52]
Haroon A et al., 2014	Eastern Med	Pakistan **	326	124	Preterms only	6				6	7	[53]
Helal NF et al., 2019	Eastern Med	Egypt **	972	674	Term and near-term		81		8	81	7	[54]
Ibekwe RC et al., 2012	African	Nigeria **	1374	237	All	40			7	40	8	[55]
Iqbal BJ et al., 2016	Eastern Med	Pakistan **	1323	377	All				15	15	5	[56]
Isa HM et al., 2017	Eastern Med	Bahrain ^++^	2940	1129	All	49		11		49	5	[57]
Israel-Aina et al., 2012	African	Nigeria **	1784	472	All	166			60	166	7	[58]
Jajoo M et al., 2019	SE Asian	India **	1675		All	136	39			136	6	[59]
Kilicdag et al., 2014	European	Turkey ^+^	5300	529	Term-near term	33		3		33	6	[60]
Kumar MN et al., 2012	SE Asian	India **	236	48	all				1	1	5	[61]
Malla T et al., 2015	SE Asian	Nepal **	1114	481	All	29				29	8	[62]
Malik FR et al., 2016	Eastern Med	Pakistan **	4497		All				62	62	4	[63]
Mirajkar S et al., 2016	SE Asian	India **	2704	575	Term		8			8	6	[64]
Mmbaga BT et al., 2012	African	Tanzania **	5033	174	All				5	5	5	[65]
Nyangabyaki-Twesigye C et al., 2020	African	Uganda *	4840	242	All	17			7	7	8	[66]
Ochigbo SO et al., 2016	African	Nigeria **	2820	553	All	17		21	8	17	8	[67]
Ogunfowora O.B et al., 2019	African	Nigeria **	2232	645	All	4	40			40	7	[68]
Ogunlesi TA et al., 2007	African	Nigeria **	4198	722	All	87		115	42	115	7	[69]
Ogunlesi TA et al., 2011	African	Nigeria **	990	152	Term			75		75	6	[70]
Ogunlesi, TA et al., 2019	African	Nigeria **	519		All		6		6	6	4	[71]
Ojukwu JU et al., 2004	African	Nigeria **	536	61	All			1	1	1	5	[72]
Okagua J et al., 2017	African	Nigeria **	622	92	All				28	28	5	[73]
Okechukwu AA et al., 2009	African	Nigeria **	654	58	All		20		11	20	5	[74]
Onyearugha CN et al., 2014	African	Nigeria **	1196	172	All	48		5	2	48	8	[75]
Osaghae DO et al., 2013	African	Nigeria **	641	105	All				3	3	5	[76]
Pius S et al., 2017	African	Nigeria **	639	64	All	30		3	5	30	7	[77]
Poudel P et al., 2009	SE Asian	Nepal **	140	103	Preterm only	29		0		29	3	[78]
Rasul CH et al., 2010	SE Asian	Bangladesh **	1981	426	All	22		9	12	22	8	[79]
Rijal P et al., 2011	SE Asian	Nepal **	820	86	All	4				4	7	[80]
Salih SA et al., 2013	Eastern Med	Sudan *	100	46	Preterm only				6	6	5	[81]
Salas AA et al., 2008	Americas	Bolivia **	1167	362	Term-near term	78		15		78	6	[82]
Simiyu DE et al., 2003	African	Kenya **	308	106	All				24	24	5	[83]
Simiyu DE et al., 2004	African	Kenya **	533	198	Preterm only	6			121	121	3	[84]
Singh SK et al., 2016	SE Asian	India **	1175	167	All	38		10		38	8	[85]
Singla DA et al., 2017	SE Asian	India **	1970	432	Term-near term	60		10	2	60	7	[86]
Speleman K et al., 2012	European	Belgium ^++^	615	363	All			12		12	7	[87]
Tagare A et al., 2013	SE Asian	India **	1801	52	Preterms only	7			31	11	6	[88]
Taghidiri MM et al., 2008	Eastern Med	Iran **	834		All		11			11	7	[89]
Tette EMA et al., 2020	African	Ghana **	2004	155	All				12	12	5	[90]
Thangavelu K et al., 2019	European	Germany ^++^	4512	1286	All	10		12	26	26	8	[91]
Thielemans L et al., 2018	SE Asian	Thailand ^+^	2980	1946	All	212				212	8	[92]
Turner C et al., 2013	SE Asian	Thailand ^+^	952	448	All				7	7	5	[93]
Udo JJ et al., 2008	African	Nigeria **	794	153	All				8	8	5	[94]
Ugochukwu EF et al., 2002	African	Nigeria **	133		Preterm only				2	2	3	[95]
Usman F et al., 2019	African	Nigeria **	360	66	Only term		20	16		20	8	[6]
Wouda EMN et al., 2020	SE Asian	Thailand ^+^	2980	1946	All	4	35		14	35	8	[96]
Zhang F et al., 2020	West Pacific	China ^+^	26,369	673	Term and near-term	195	73			195	6	[97]

* low income country; ** lower middle income country; ^+^ upper middle income country; ^++^ high income country. WHO: World Health Organization; NNJ: neonatal jaundice; EBT: exchange blood transfusion; abnormal Brainstem Auditory Evoked Response: aBAER; acute bilirubin encephalopathy: ABE: severe neonatal jaundice: SNJ: Reference number: Ref #: Eastern Mediterranean: Eastern Med; South-East Asian: SE Asian.

**Table 2:** Prevalence (%) of severe neonatal jaundice (SNJ) among all hospital admissions.

	N	Estimates (95% Confidence Interval)	*p*-Value for Heterogeneity
Overall	84	2.55 (1.93–3.27)	-
Country Income Level			0.013
High	6	0.81 (0.11–2.09)	
Upper-middle	14	1.76 (1.00–2.73)	
Lower-middle	59	3.15 (2.25–4.18)	
Low	5	1.47 (0.36–3.20)	
Gestation			0.389
Preterm	8	6.28 (1.68–13.32)	
Term and near term	14	2.22 (1.11–3.71)	
Term	8	2.04 (0.49–4.57)	
All	54	2.36 (1.71–3.10)	
Quality of Study			0.065
High	43	2.83 (2.02–3.78)	
Moderate	34	1.73 (1.05–2.57)	
Low	7	5.90 (1.44–12.97)	

N: Number of studies; References: [6,15,16,17,18,19,20,21,22,23,24,25,26,27,28,29,30,31,32,33,34,35,36,37,38,39,40,41,42,43,44,45,46,47,48,49,50,51,52,53,54,55,56,57,58,59,60,61,62,63,64,65,66,67,68,69,70,71,72,73,74,75,76,77,78,79,80,81,82,83,84,85,86,87,88,89,90,91,92,93,94,95,96,97].

**Table 3:** Prevalence (%) of neonatal jaundice (NNJ) among all hospital admissions by World Health Organization (WHO) region.

WHO Regions ^a^	N	Estimates (95% Confidence Interval)
Overall	74	21.99 (18.42–25.78)
African	34	20.10 (16.06–24.47)
Eastern Mediterranean	4	16.66 (4.90–33.54)
European	10	20.39 (11.73–30.70)
Americas	4	13.13 (2.83–29.34)
South-East Asian	21	30.61 (22.19–39.74)
Western Pacific	1	2.55 (2.37–2.75)

N: Number of studies; WHO: World Health Organization. ^a^ Test for subgroup differences: *p*-value < 0.0001. References: [6,15,16,18,19,20,22,23,24,25,26,27,28,29,30,31,32,33,34,36,37,38,39,40,41,42,43,44,45,47,48,49,50,51,52,53,54,55,56,58,60,61,62,64,65,66,67,68,69,70,72,73,74,75,76,77,78,79,80,81,82,83,84,85,86,87,88,90,91,92,93,94,96,97].

**Table 4:** Prevalence (%) of severe neonatal jaundice (SNJ) and clinical markers among *all hospitalized neonates* by World Health Organization (WHO) region.

	African		Eastern Mediterranean		European		South-East Asian		Americas		Western Pacific	
	N	Estimates (95% CI)	N	Estimates (95% CI)	N	Estimates (95% CI)	N	Estimates (95% CI)	N	Estimate (95% CI)	N	Estimates (95% CI)
SNJ ^a^	39	3.34 (2.28–4.57)	5	1.42 (0.93–2.02)	10	1.31 (0.61–2.27)	24	2.58 (1.33–4.22)	5	1.73 (0.14–4.92)	1	0.74 (0.64–0.85)
EBT ^b^	16	3.81 (2.14–5.92)	3	1.19 (0.80–1.66)	9	1.25 (0.51–2.30)	17	3.50 (1.69–5.90)	3	2.64 (0.17–7.71)	1	0.74 (0.64–0.85)
ABE ^c^	19	2.75 (1.75–3.95)	2	1.02 (0.05–3.16)	8	0.16 (0.02–0.40)	6	0.83 (0.36–1.46)	2	0.34 (0.00–2.72)	1	0.28 (0.22–0.34)
Jaundice Related Death ^d^	35	1.49 (0.85–2.28)	2	1.24 (0.00–4.48)	3	0.01 (0.00–0.04)	11	0.82 (0.27–1.62)	1	0.00 (0.00–0.00)	-	-

ABE: Acute Bilirubin Encephalopathy; CI: Confidence interval; EBT: Exchange Blood Transfusion; N: Number of studies; SNJ: Severe neonatal jaundice; WHO World Health Organization. ^a^ Test for subgroup differences: *p*-value < 0.001. ^b^ Test for subgroup differences: *p*-value < 0.001. ^c^ Test for subgroup differences: *p*-value < 0.001. ^d^ Test for subgroup differences: *p*-value < 0.001 References: [6,15,16,17,18,19,20,21,22,23,24,25,26,27,28,29,30,31,32,33,34,35,36,37,38,39,40,41,42,43,44,45,46,47,48,49,50,51,52,53,54,55,56,57,58,59,60,61,62,63,64,65,66,67,68,69,70,71,72,73,74,75,76,77,78,79,80,81,82,83,84,85,86,87,88,89,90,91,92,93,94,95,96,97].

**Table 5:** Prevalence (%) of severe neonatal jaundice (SNJ) and clinical markers *among hospitalized neonates with jaundice* by World Health Organization (WHO) region.

	African		Eastern Mediterranean		European		South-East Asian		Americas		Western Pacific	
	N	Estimates (95% CI)	N	Estimates (95% CI)	N	Estimates (95% CI)	N	Estimates (95% CI)	N	Estimate (95% CI)	N	Estimates (95% CI)
SNJ	34	18.39 (12.87–24.63)	4	12.58 (3.40–26.28)	10	9.02 (2.64–18.62)	21	8.31 (4.20–13.60)	4	31.49 (0.00–89.12)	1	28.97 (25.61–32.46)
EBT ^a^	14	21.42 (11.03–34.07)	3	12.13 (1.09–32.11)	9	9.76 (2.57–20.80)	15	10.86 (5.32–18.01)	2	17.03 (9.62–26.02)	1	28.97 (25.61–32.46)
ABE ^b^	17	14.51 (9.08–20.90)	2	22.73 (0.00–91.81)	8	2.01 (0.00–8.10)	5	2.07 (0.85–3.74)	2	1.46 (0.00–7.94)	1	10.85 (8.60–13.31)
Jaundice Related Death ^c^	31	7.52 (4.95–10.56)	2	13.02 (9.64–16.81)	3	0.07 (0.00–0.20)	10	2.01 (1.06–3.20)	-	-	-	-

ABE: Acute Bilirubin Encephalopathy; aBAER: Abnormal Brainstem auditory evoked response; CI: Confidence interval; EBT: Exchange Blood Transfusion; N: Number of studies; SNJ: severe neonatal jaundice; WHO World Health Organization. ^a^ Test for subgroup differences: *p*-value < 0.001. ^b^ Test for subgroup differences: *p*-value < 0.001. ^c^ Test for subgroup differences: *p*-value < 0.001. References: [6,15,16,16,17,18,19,20,22,23,24,25,26,27,28,29,30,31,32,33,34,36,37,38,39,40,41,42,43,44,45,47,48,49,50,51,52,53,54,55,56,58,60,61,62,64,65,66,67,68,69,70,72,73,74,75,76,77,78,79,80,81,82,83,84,85,86,87,88,90,91,92,93,94,96,97].

**Table 6:** Prevalence (%) of severe neonatal jaundice (SNJ) among all hospital admissions according to methodological domains for assessing quality of study.

	N	Estimates (95% Confidence Interval)	*p* Value for Test for Subgroup Differences
Overall	84	2.55 (1.93–3.27)	-
Sample representative of target population			0.329
All	57	2.39 (1.76–3.10)	
Term and near term	16	2.03 (1.04–3.32)	
Term only or preterm only	11	4.85 (1.39–10.11)	
Method used to define jaundice			0.749
Serum bilirubin	52	2.70 (1.92–3.60)	
Clinically	3	1.80 (0.27–4.56)	
Not stated	29	2.39 (1.30–3.77)	
Study excludes any of the following: G6PD, ABOi, Rhi, Sepsis			0.055
Yes	4	1.20 (0.33–2.59)	
No	80	2.64 (1.98–3.39)	
Study reported total number of NNJ cases			0.040
Yes	74	2.72 (2.00–3.54)	
No	10	1.56 (0.90–2.39)	
Was clinically significant jaundice clearly defined in methods (including use of AAP/NICE. etc guidelines)?			0.646
Yes	45	2.42 (1.67–3.30)	
No	39	2.71 (1.71–3.93)	
Type of healthcare facility			<0.001
Tertiary/referral	68	3.01 (2.25–3.86)	
Secondary, PHC, community	2	0.62 (0.28–1.08)	
Not stated	14	1.06 (0.38–2.06)	

AAP: American Academy of Pediatrics; ABOi: ABO-incompatibility; G6PD: Glucose-6-phosphate dehydrogenase; N: Number of studies; nice: National Institute of Health and Care Excellence; NNJ: Neonatal jaundice; PHC: primary health care; References: [6,15,16,17,18,19,20,21,22,23,24,25,26,27,28,29,30,31,32,33,34,35,36,37,38,39,40,41,42,43,44,45,46,47,48,49,50,51,52,53,54,55,56,57,58,59,60,61,62,63,64,65,66,67,68,69,70,71,72,73,74,75,76,77,78,79,80,81,82,83,84,85,86,87,88,89,90,91,92,93,94,95,96,97].

**Table 7:** Univariate mixed effects meta-regression analysis relating study-level factor and methodological domains for assessing quality of study to the proportion of severe neonatal jaundice (SNJ) among all hospital admissions.

	Estimates (95% Confidence Interval)	Heterogeneity Accounted for by Factor	*p* Value
Year of publication	−0.006 (−0.010, −0.002)	24.97%	<0.001
Country income level		50.99%	0.009
High	Referent		
Upper-middle	0.041 (−0.028, 0.111)		
Lower-middle	0.087 (0.025, 0.149)		
Low	0.036 (−0.056, 0.153)		
Sample representative of target population		45.40%	0.040
Term only or preterm only	Referent		
Term and near term	−0.075 (−0.136, −0.013)		
All	−0.062 (−0.114, 0.010)		
Method used to define jaundice		8.39%	0.831
Not stated	Referent		
Clinically	−0.021 (−0.140, 0.097)		
Serum bilirubin/AAP/NICE	0.009 (−0.036, 0.055)		
Study excludes any of the following: G6PDd, ABOi, Rhi, Sepsis		0.00%	0.303
Yes	Referent		
No	0.055 (−0.050, 0.159)		
Study reported total number of NNJ cases		14.68%	0.219
No	Referent		
Yes	0.040 (−0.024, 0.104)		
Was clinically significant jaundice clearly defined in methods?		28.77%	0.621
No	Referent		
Yes	−0.010 (−0.048, 0.029)		
Type of healthcare facility			
Not stated	Referent	59.73%	0.001
Secondary, PHC, community	−0.024 (−0.124, 0.077)		
Tertiary/referral	0.070 (0.031, 0.109)		

AAP: American Academy of Pediatrics; NICE: National Institute for Healthcare and Excellence; G6PDd: Glucose-6-phosphate dehydrogenase deficiency; ABOi: ABO incompatability; Rhi: Rhesus incompatability; NNJ neonatal jaundice; PHC: Primary Healthcare Center.

## Data Availability

Not applicable.

## Supplementary Materials

The following supporting information can be downloaded at: https://www.mdpi.com/article/10.3390/jcm12113738/s1.
